# Checkpoint activation drives global gene expression changes in Drosophila nuclear lamina mutants

**DOI:** 10.1093/g3journal/jkab408

**Published:** 2021-12-10

**Authors:** Samuel Cole Kitzman, Tingting Duan, Miles A Pufall, Pamela K Geyer

**Affiliations:** Department of Biochemistry, University of Iowa, Iowa City, IA 52242, USA

**Keywords:** nuclear lamina, LEM-domain proteins, Drosophila oogenesis, germline stem cells, emerin, Checkpoint kinase 2, Chk2, Barrier-to-autointegration factor

## Abstract

The nuclear lamina (NL) lines the inner nuclear membrane. This extensive protein network organizes chromatin and contributes to the regulation of transcription, DNA replication, and repair. Lap2-emerin-MAN1 domain (LEM-D) proteins are key members of the NL, representing proteins that connect the NL to the genome through shared interactions with the chromatin-binding protein Barrier-to-Autointegration Factor (BAF). Functions of the LEM-D protein emerin and BAF are essential during *Drosophila melanogaster* oogenesis. Indeed, loss of either emerin or BAF blocks germ cell development and causes loss of germline stem cells, defects linked to the deformation of NL structure, and non-canonical activation of Checkpoint kinase 2 (Chk2). Here, we investigate the contributions of emerin and BAF to gene expression in the ovary. Profiling RNAs from *emerin* and *baf* mutant ovaries revealed that nearly all *baf* misregulated genes were shared with *emerin* mutants, defining a set of NL-regulated genes. Strikingly, loss of Chk2 restored the expression of most NL-regulated genes, identifying a large class of Chk2-dependent genes (CDGs). Nonetheless, some genes remained misexpressed upon Chk2 loss, identifying a smaller class of emerin-dependent genes (EDGs). Properties of EDGs suggest a shared role for emerin and BAF in the repression of developmental genes. Properties of CDGs demonstrate that Chk2 activation drives global misexpression of genes in the *emerin* and *baf* mutant backgrounds. Notably, CDGs were found upregulated in *lamin-B* mutant backgrounds. These observations predict that Chk2 activation might have a general role in gene expression changes found in NL-associated diseases, such as laminopathies.

## Introduction

The nuclear lamina (NL) is an extensive protein network composed of lamins and lamin-associated proteins that lies beneath the inner nuclear membrane. This network plays a central role in establishing the three-dimensional structure of the genome, a feature important for transcription, DNA replication, and repair (Goldman *et al.* 2002; Geyer *et al.* 2011; Gonzalo 2014). Indeed, large regions of metazoan genomes associate with the NL (van Steensel and Belmont 2017). These lamina-associated domains (LADs) display properties typical of heterochromatin, including low or no expression of resident genes. Transcriptional activation of LAD genes often requires gene movement away from the NL and into the nuclear interior (Shevelyov *et al.* 2009; Kohwi *et al.* 2013; Czapiewski *et al.* 2016; Brueckner *et al.* 2020), promoting concepts that NL proximity imparts transcriptional repression (Briand and Collas 2020). Changes in contacts between the NL and genome occur during differentiation (Peric-Hupkes and van Steensel 2010; Kohwi *et al.* 2013; Chen *et al.* 2014; Czapiewski *et al.* 2016; Flint Brodsly *et al.* 2019), indicating that NL association participates in the developmental regulation of transcription. Dysfunction of NL components causes age-enhanced human diseases known as laminopathies (Zink *et al.* 2004; Scaffidi and Misteli 2006; Loi *et al.* 2016). These diseases weaken the NL network and alter nuclear stability. Consequently, contacts between the NL and the genome are disrupted, leading to global changes in nuclear architecture and transcription (Czapiewski *et al.* 2016; Osmanagic-Myers and Foisner 2019). Together, these data demonstrate that NL association is important for regulated transcription.

Proteins required for NL function are cell type specific. For example, lamins are not essential for organizing the genome in all cell types, illustrated by findings that removal of all lamins in mouse embryonic stem cells only minimally perturbs interactions between the genome and NL (Amendola and van Steensel 2015). These observations imply that non-lamin components contribute to building nuclear architecture. One key NL protein family that promotes genome organization is the Lap2-emerin-MAN domain (LEM-D) family (Brachner and Foisner 2011; Barton *et al.* 2015). LEM-D proteins bridge the NL and genome through shared interactions with Barrier-to-Autointegration Factor (BAF, sometimes referred to as BANF1), a histone, lamin, and sequence-independent double-strand DNA-binding protein (Berk *et al.* 2013; Kind and van Steensel 2014). Support for the contributions of LEM-D proteins to nuclear architecture comes from studies of family members such as emerin, a LEM-D protein that contributes to the spatial and temporal regulation of genes during differentiation (Demmerle *et al.* 2013; Koch and Holaska 2014). Such observations indicate that LEM-D proteins are important proteins in the NL network that contribute to regulated gene expression.

Studies in Drosophila have advanced our understanding of developmental roles of LEM-D proteins (Barton *et al.* 2015; Duan *et al.* 2020a). Drosophila has four LEM-D proteins (Pinto *et al.* 2008), of which three localize to the NL (Wagner *et al.* 2006). These include two emerin orthologs (emerin also known as Otefin and emerin2 also known as Bocksbeutel), as well as Drosophila MAN1 (dMAN1; Wagner and Krohne 2007). The best characterized Drosophila LEM-D protein is emerin, a protein required for female and male fertility (Jiang *et al.* 2008; Barton *et al.* 2013; Barton *et al.* 2016). Infertility in *emerin* mutants is linked to germline stem cell (GSC) loss caused by structural deformation of the NL that non-canonically activates two DNA damage response kinases, Ataxia Telangiectasia and Rad3-related (ATR), and Checkpoint kinase 2 (Chk2; Barton *et al.* 2018). Germ-line knockdown (GLKD) of BAF also causes GSC loss due to NL deformation and checkpoint kinase activation (Duan *et al.* 2020b). Taken together, these findings emphasize the importance of NL integrity for the health and longevity of adult stem cells and tissue homeostasis.

How NL changes affect adult stem cell homeostasis is unclear. Here, we investigate how changes in the NL structure affect gene expression. To this end, we profiled RNAs isolated from *emerin* and *baf* GLKD mutant ovaries. RNA sequencing (RNA-seq) revealed that thousands of genes were misexpressed in these *NL* mutant backgrounds. Nearly all *baf* misregulated genes were shared with *emerin* mutants, defining a set of NL-regulated genes. To understand how checkpoint activation contributed to these expression changes, we profiled RNA isolated from *chk2, emerin* double mutant ovaries. Strikingly, loss of Chk2 restored the expression of nearly all genes misregulated in *emerin* mutants, demonstrating that Chk2 drives altered gene expression in *NL* mutants. Even so, a small class of emerin-dependent genes (EDGs) remained, along with a larger class of Chk2-dependent genes (CDGs). Properties of EDGs support a shared role for emerin and BAF in the repression of developmental genes. Properties of CDGs indicate that Chk2 drives global misexpression of genes upon activation by NL deformation. Strikingly, CDGs represent over a third of genes upregulated upon somatic knockdown of Lamin-B, providing evidence that Chk2 activation might broadly contribute to gene expression changes resulting from NL dysfunction. Taken together, our studies suggest that activated Chk2 might contribute to gene expression changes found in NL diseases, such as laminopathies.

## Materials and methods

### Drosophila stocks and culture conditions

Drosophila stocks were raised on standard cornmeal/agar medium with *p*-hydroxybenzoic acid methyl ester as a mold inhibitor. All crosses were carried out at 25°C and 70% humidity. Two reference strains were used, including *y^1^, w^67c23^* (*yw*) and *Canton S*. Three *emerin* (*otefin, ote*) null alleles were used, all in an *yw* mutant background. These included *ote^G^* (also known as *ote^B279G^*) that carries an insertion of a Piggybac transposon at +764, *ote^PK^* that carries an R127term mutation and *ote^PI^* that carries a V413D amino acid substitution (Barton *et al.* 2013). Two *chk2* (*loki*) double mutant stocks were used, including *yw; chk2^P6^*, *ote^PK^/y^+^CyO* (R27) wherein *chk2^P6^* carries a *P* insertion and deletion affecting the second exon (Abdu *et al.* 2002) and *yw; chk2^P30^*, *ote^G^/y^+^CyO* (R13) wherein *chk2^P30^* carries a deletion of the 5′UTR and first two exons (LaRocque *et al.* 2007). GLKD of BAF was achieved crossing the *w^1118^, nanos (nos)>gal4VP16* driver line (Bloomington # 4937) and the y^1^, sc*, v^1^, sev^21^; P[Trip.HMS00195]attP2 (Bloomington # 36108) short hairpin RNA responder. Other mutants used include *tud^1^* that carries a K1036term mutation, *tud^A36-38^* (a kind gift of the R. Lehmann laboratory), and *bag-of-marbles* (*bam^Δ86^*; BL # 5427) that carries a 1.7-kb deletion in the coding region (Bopp *et al.* 1993).

### Immunohistochemical analyses

For phenotypic analyses, ovaries were dissected into cold phosphate-buffered saline (PBS) solution and immediately fixed at room temperature in 4% EM Grade paraformaldehyde (Electron Microscopy Sciences no. 15710). Fixed ovaries were washed in PBS with 0.3% Triton-X 100 (PBST), blocked in PBTS with 5% w/v BSA at room temperature for 1 h and incubated at 4°C with primary antibody overnight. Next, ovaries were incubated with Alexa Fluor-conjugated secondary antibodies (Molecular Probes) at room temperature for 2 h, washed in PBST, stained with 1 µg/ml DAPI (ThermoFisher scientific), and mounted in SlowFade (ThermoFisher). Images were collected with a Zeiss 710 confocal microscope and processed using ZEN imaging software. Primary antibodies included: (1) mouse α-Lamin at 1:300 (DSHB ADL84.12), (2) mouse α-Engrailed at 1:100 (DSHB, 4D9), and (3) rabbit α-Vasa at 1:300 (Santa Cruz Biotechnology). Experiments were performed with two to three biological replicates of at least five pairs of ovaries per experiment.

### RNA isolation and analysis of gene expression

RNA-seq was completed to obtain a genome-wide view of gene expression changes caused by NL dysfunction. RNA was isolated from ovaries dissected from 4- to 6-h-old females. Ovaries of this age are developmentally immature and lack the transcriptionally active late-stage egg chambers (Klug *et al.* 1968), a strategy used to enrich for RNA production emanating from cells of the stem cell niche. Ovaries were dissected in PBS on ice and stored at −80°C. RNA was isolated with TRIzol (Invitrogen) extraction and treated with DNase I and column purified with the RNAeasy kit (Qiagen). In total, RNA-seq was completed on independent biological RNA isolates from (1) three *CS*; (2) three *yw*; (3) two *nosgal4* (driver only); (4) two *emerin^G/PK^*; (5) two *emerin^G/PI^*; (6) two *baf GLKD*; and (7) three *chk2^P6/P30^*, *emerin^G/PK^*. All RNA samples were sent to Novogene for library preparation involving polyA capture to remove rRNA and reverse transcription prior to sequencing. Illumina PE150 technology was used to sequence the samples. Downloaded sequencing results were converted to FASTQ format and adaptor sequences were trimmed from the paired reads using Trim-Galore! (v0.0.6) in paired mode. Trimmed sequences were aligned to the *Drosophila* *melanogaster* reference transcriptome (version dm6; BDGP6.32 from Ensembl) using Kallisto (v0.46.1) with bootstraps set to 30. Transcript level counts were joined and converted to gene level counts using the tximeta package in R (v1.6.3). Differential expression analysis was performed using DESeq2 (v1.28.1) in R, with significant genes defined as genes with an adjusted *P*-value <0.05. Genes with fewer than 10 total counts across all genotypes were excluded from analysis. Principal component analysis (PCA) plots were generated in R using DESeq2 (v1.28.1) along with ggplot (v1.6.3).

Overlaps between differentially expressed gene sets identified in *NL* mutants (Supplementary Table S1) and published datasets were completed in R using BioVenn (v1.1.2). These analyses included differentially expressed genes identified in the analysis of *tud* mutant ovaries (Coux *et al.* 2018; GSE108473 supplemental file GSE108472_Soma_RNA seq-1), *bam* mutant ovaries (Tiwari *et al.* 2019; Supplementary Table S2, GSE138987), Lamin-B depletion in Kc cells (Nazer *et al.* 2018; Supplementary Table S2, GSE95844), and Lamin-B depletion in the fat body (Chen *et al.* 2014; Supplementary Table S4, GSE62580). The statistical significance of overlaps between gene sets was determined using Fisher’s exact tests (FETs), completed in the R package GeneOverlap (v1.24.0). To define the chromatin profile of *NL* misregulated genes, a GTF file containing coordinates of each of the five chromatin states (Filion *et al.* 2010) was mapped to the dm6 genome (FlyBase), using the intersect command from bedtools (v2.29.2). The generated BED file with gene transcripts linked to a chromatin color was filtered in R to ensure that the assigned chromatin color was the color associated with the gene TSS, an approach used previously (Filion *et al.* 2010). Genes with multiple promoters in different chromatin colors were randomly assigned to a single color. Pathway analyses used FlyEnrichr, an analysis tool built from 35 Drosophila libraries (Kuleshov *et al.* 2019). FlyEnrichr was used for specific KEGG pathway and transcription factor target identification.

Quantitative PCR used RNA isolated from ∼50 ovary parts. RNA was converted into cDNA using the High Capacity cDNA kit with random hexamer primers (Applied Biosystems). Primers used in this study are listed in Supplementary Table S2. Cycle threshold levels were normalized to the housekeeping gene, *RpL32*, and fold-enrichment was calculated relative to the *yw* reference strain using the ΔΔCt method (Livak and Schmittgen 2001).

## Results and discussion

### NL dysfunction causes gene expression changes in the ovary

**Figure 1:**
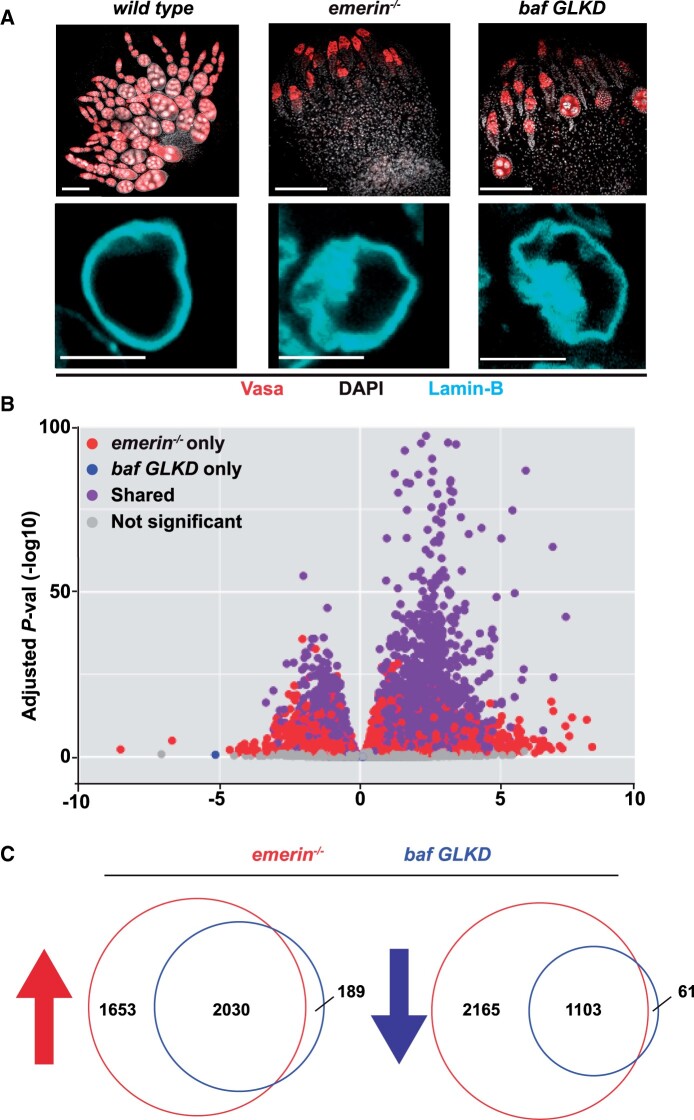
Shared gene expression changes in *emerin* and *baf* mutant ovaries. (A) Top: shown are representative confocal images of ovaries dissected from newly born *wild-type*, *emerin^−/−^*, and *baf* germline knockdown (*baf* GLKD) females stained with an antibody against Vasa (red) and DAPI (white). Scale bar: 100 μm. Bottom: confocal images of individual GSC nuclei stained with an antibody against Lamin-B (blue). Scale bar: 5 μm. (B) Volcano plot of gene expression changes in *emerin* and *baf* GLKD mutants, showing expression changes between *emerin* (red) or *baf* GLKD (blue) and wild-type references, with overlap between these datasets indicated in purple. (C) Venn diagrams of up- and downregulated genes in *emerin* (red) mutant and *baf GLKD* (blue) relative to wild-type references.

Survival of adult stem cells in the female and male germlines depends upon emerin and BAF (Barton *et al.* 2013, 2016; Duan *et al.* 2021). To gain further insights into the mechanism of stem cell loss, we investigated gene expression changes in the ovary resulting from loss of either NL component. We focused on the ovary, as both emerin and *baf* mutant phenotypes are present in newborn *emerin* and *baf* GLKD mutant females (Figure  1A) but have an age-dependent component in males (Barton *et al.* 2016). Ovaries of newly born females are developmentally immature and lack the transcriptionally active later stages of oogenesis (Klug *et al.* 1968). As such, these ovaries provided a strategy to define the RNA profile of cells in the stem cell niche.

First, we defined effects on the gene expression of emerin loss. We performed RNA-seq of RNAs isolated from ovaries of newly born females of two wild-type reference strains (*Canton S* and *yw*) and two different *emerin* null backgrounds (*emerin^G/PK^* and *emerin^G/PI^*). Two reference and two *emerin* mutant backgrounds were used to minimize the identification of bystander genes for which expression changes were unrelated to emerin loss. The GSC loss phenotype is manifest in newly born *emerin* mutant ovaries (Figure  1A), ranging from a median of 18% of germaria without GSCs in *emerin^G/PK^* ovaries to 56% of germaria without GSCs in *emerin^G/PI^* ovaries (Barton *et al.* 2013). Comparison of RNA-seq data from the two reference and *emerin* mutants identified large numbers of genes with significantly increased (3683, adj *P*-value ≤0.05) and decreased (3268, adj *P*-value ≤0.05) expression in *emerin* mutants relative to the reference strains (Figure  1, B and C and Supplementary Table S1). To determine whether loss of emerin caused compensatory changes in other NL genes, we mapped RNA-seq reads to individual NL genes. These analyses revealed no consistent changes in the expression of *emerin2*, *dMAN1*, or *baf* upon emerin loss (Supplementary Figure S1). Based on these data, we conclude that loss of the single NL component emerin causes widespread disruption of gene expression.

Second, we defined effects on gene expression of germ cell-specific loss of BAF. We completed RNA-seq of RNAs obtained from ovaries of newly born females carrying only the driver transgene (*nosgal4*) and females carrying both the driver and responder transgenes (*baf* GLKD). Extensive GSC loss is observed in newly born *baf* GLKD ovaries, with a median of 38% of germaria without GSCs (Figure  1A) (Duan *et al.* 2020b). Comparison between *nosgal4* and *baf* GLKD transcriptomes identified 2219 genes with significantly increased (adj *P*-value ≤0.05) and 1164 genes with significantly decreased (adj *P*-value ≤0.05) expression (Figure  1, B and C and Supplementary Table S1). As expected from the shared phenotypes (Figure  1A), the *emerin* and *baf* mutant backgrounds show strong overlap between misregulated genes. Notably, 91% (2030) of *baf* upregulated and 95% (1103) of *baf* downregulated genes were also identified in *emerin* mutants (Figure  1, B and C), defining a set of NL-regulated genes. These data indicate that the emerin and BAF partnership extends to the regulation of gene expression in the ovary.

Third, we performed gene ontology and pathway analysis of the NL-regulated gene set. Loss of emerin and BAF causes activation of a DNA damage response (Barton *et al.* 2018). Motivated by this finding, we manually inspected the expression of high confidence irradiation-induced DNA damage response genes in reference and *NL* mutant backgrounds. This inspection identified only three shared genes [8% (3/38), *P = *0.92; van Bergeijk *et al.* 2012], including Jun-related antigen (Jra), Kayak (Kay), and CHKov1. These findings reinforce observations that ATR and Chk2 activation in *emerin* mutants is independent of DNA damage (Barton *et al.* 2018). Strikingly, Jra and Kay are subunits of Drosophila AP-1, a downstream effector of the stress-induced Jun N-terminal kinase (JNK) pathway (Herrera and Bach 2021). As JNK activation is linked to apoptosis, we suggest that JNK activation might contribute to GSC loss in *emerin* and *baf* mutant backgrounds. Next, we used FlyEnrichr to understand gene pathways identified in the NL-regulated genes (Kuleshov *et al.* 2019). These enrichment analyses identified the Toll and Imd pathways as significant among upregulated genes (Table  1), two pathways involved in the regulation of the Drosophila immune response (De Gregorio *et al.* 2002; Tanji *et al.* 2007). Enrichment analyses of downregulated genes identified components of translation, DNA replication, and DNA repair pathways as significant (Table  1), an outcome consistent with the absence of developing oocytes (Figure  1A). Taken together, the gene expression profile resulting from NL dysfunction shows the evidence of stress-induced cell death associated with blocked oogenesis.

**Table 1:** KEGG pathway analysis of shared emerin and BAF misregulated genes

Pathway	Adjusted *P*-Value
Upregulated genes (1992)	
Toll and Imd signaling	1.9e−4
Downregulated genes (1051)	
AA-tRNA biosynthesis	1.5e−18
Nucleotide excision repair	1.3e−9
RNA degradation	9.7e−8
DNA replication	2.0e−6
Dorsal ventral axis formation	9.0e−5

### *NL* mutants share gene expression changes with other GSC mutants

**Figure 2:**
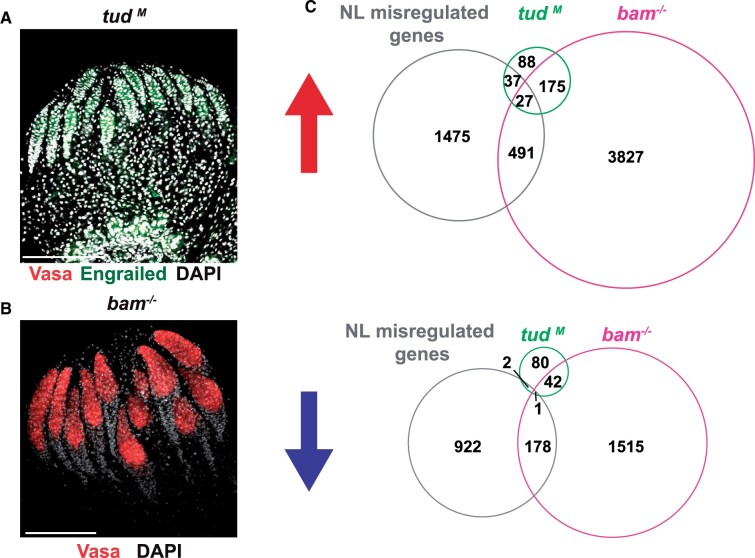
*NL* mutants alter gene expression in the soma and germline. (A) Shown is a representative confocal image of a newly born *tudor* maternal mutant (*tud^M^*) ovary stained with antibodies against Vasa (red), Engrailed (green), and DAPI. Scale bar: 100 μm. (B) Shown is a representative confocal image of a newly born *bam* mutant (*bam^−/−^*) ovary stained with an antibody against Vasa (red) and DAPI. Scale bar: 100 μm. (C) Venn diagrams of NL shared genes overlapped with up- or downregulated genes in *tud* (green; Coux *et al.* 2018) and *bam* (red; Tiwari *et al.* 2019) mutant backgrounds relative to wild-type references.

The set of NL-regulated genes was derived from shared gene expression changes resulting from global loss of emerin, but germ cell restricted loss of BAF. As such, this strategy emphasizes consequences of NL dysfunction in germ cells. Even so, we reasoned that the set of NL-regulated genes likely included genes with expression changes in somatic and germ cells. To understand the contributions from these different cell types, we assessed whether *NL* mutants produce common gene expression signatures with GSC homeostasis mutants that lack or over-proliferate GSCs (Yan *et al.* 2014). First, we compared the set of NL-regulated genes with genes misexpressed in *tud* mutants (Supplementary Table S1; Coux *et al.* 2018). Tudor is required for germ cell specification (Thomson and Lasko 2004), such that all *tud* mutant ovaries completely lack germ cells (Figure  2A). As such, overlapping changes in gene expression are expected to address how germ cell NL dysfunction affects somatic cells. Notably, significant overlap was observed between *NL* and *tudor* upregulated (3%, 64/2030, *P < *0.041) but not downregulated genes (0.3%, 3/1103, *P *=* *1; Figure  2C). These data support that the NL upregulated genes include expression changes in the soma. Second, we compared the set of NL-regulated genes with genes misexpressed in *bam* mutant GSCs. Bam is essential for GSC differentiation due to an requirement in the regulation of mRNAs encoding stem cell maintenance factors. Loss of Bam results in ovaries that carry tumors of GSC-like cells (Figure 2B; Tiwari *et al.* 2019). In this case, overlapping changes in gene expression are expected to address germ cell consequences of the NL-regulated genes. These comparisons revealed no significant overlap between *NL* and *bam* upregulated genes (36%, 518/2030, *P *=* *1) but significant overlap with downregulated genes (16%, 179/1103, *P *<* *4.9e−3; Figure  2C). These data support that the NL downregulated genes represent differentiation gene changes in the germ cell. Taken together, these findings provide evidence that shared defects in the *NL* mutants integrate gene expression changes in somatic and germ cells.

### Chk2 activation has a major role in gene expression changes in *emerin* mutants

**Figure 3:**
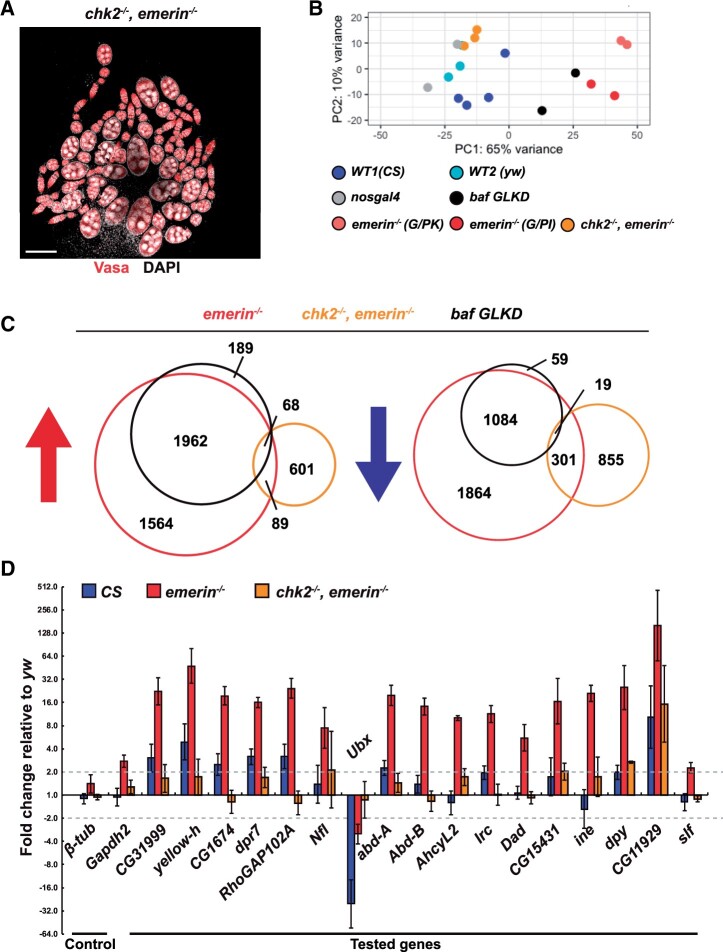
Loss of Chk2 largely restores ovary gene expression changes in *emerin* mutants. (A) Shown is a representative confocal image of a newly born *chk2*, *emerin* double mutant (*chk2^p6p30^*, *emerin^G/PK^*) ovary stained with an antibody against Vasa (red) and DAPI (white). Scale bar: 100 μm. (B) PCA of normalized RNA-seq data obtained from newly born ovaries dissected from three different *wild-type* (*WT*) females (*CS*, *yw*, and *nosgal4*), two *emerin* mutant females (*G/PK* and *G/PI*), *baf* GLKD females and *chk2*, *emerin* double mutant females (*chk2^p6p30^*, *emerin^G/PK^*). (C) Venn diagrams showing overlaps between up- and downregulated genes identified in *emerin*, *baf* and *chk2*, *emerin* double mutants relative to wild-type reference controls. (D) Quantitative RT-PCR of RNAs isolated from newly born ovaries of *WT* (CS, blue), *emerin* mutants (*emerin^G/PK^*, red), *chk2, emerin* double mutants (*chk2^p6p30^*, *emerin^G/PK^*, green). RNAs were normalized to levels of the housekeeping *RpL32* gene and fold change is shown relative to RNA levels in *yw.*

Oogenesis is restored in *chk2, emerin* double mutants (Figure  3A). Indeed, newly born double mutant ovaries show no GSC loss and females lay wild-type levels of eggs for days (Barton *et al.* 2018). We reasoned that RNA-seq analyses of *chk2, emerin* double mutant ovaries would advance our understanding of how activated Chk2 shapes gene expression in the *NL* mutant backgrounds. To this end, we completed RNA-seq on triplicate biological replicates of RNA obtained from ovaries isolated from newly born *chk2, emerin* double mutant females. PCA and unsupervised clustering of RNA-seq data showed that *chk2, emerin* double mutants clustered with wild-type backgrounds but were separate from *emerin* or *baf* mutant backgrounds (Figure  3B and Supplementary Figure S2). These observations provide molecular evidence that loss of Chk2 strongly suppresses the *emerin* mutant phenotype. In total, we identified 758 upregulated and 1175 downregulated genes in *chk2, emerin* double mutants relative to expression in the wild-type references (Figure  3C and Supplementary Table S1). To determine the degree of rescue, the set of *chk2, emerin* misregulated genes was overlapped with *emerin* misregulated genes. Strikingly, a near universal restoration of expression occurred, as only 4% (157/3683) of upregulated and 10% (320/3268) of downregulated genes found in *emerin* mutants remained dysregulated in *chk2, emerin* double mutants (Figure  3C). To substantiate the extensive rescue by the loss of Chk2, expression of 17 randomly selected NL upregulated genes was directly quantified in RNAs isolated from two wild-type reference strains, *emerin* mutants and *chk2, emerin* double mutants. In all but one case (94%; 16/17), loss of emerin increased RNA levels of the test gene (Figure  3D). As expected from the RNA-seq data, loss of Chk2 restored expression to genes upregulated in *emerin* mutants. Building from the overlap of *emerin* and *chk2, emerin* RNA-seq data, we identified two classes of misregulated genes in *emerin* mutants, a small class that remains misexpressed in the *chk2, emerin* double mutant (157 upregulated and 320 downregulated; EDGs) and a large class that shows restored expression in the *chk2, emerin* double mutant (3526 upregulated and 2948 downregulated; CDGs). Based on these data, we conclude that Chk2 is the main driver of altered gene expression in the *emerin* mutants.

### Expression of upregulated EDGs depends on BAF

Hundreds of EDGs were misregulated in *chk2, emerin* double mutants (Figure  3C). Nearly half (43%, 68/157, *P *<* *8.1e−17) of upregulated EDGs are shared with *baf* GLKD, whereas only 6% (19/320, *P < *0.95) of downregulated are shared (Figure  3C). Based on these findings, we conclude that the emerin and BAF partnership is primarily used for the repression of target genes.

**Figure 4:**
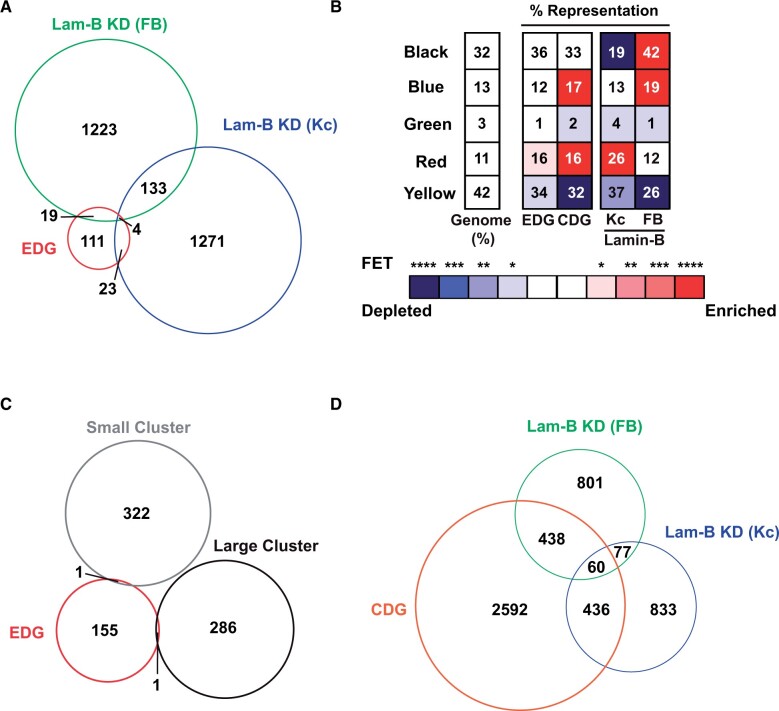
Features of EDGs and CDGs. (A) Venn diagrams showing overlap of up- and downregulated EDGs with misregulated genes resulting from Lamin-B knockdown in Kc cells (Nazer *et al.* 2018) and in the adult fat body (FB; Chen *et al.* 2014). (B) Shown is a heat map of enrichment or depletion across the five chromatin colors for upregulated EDGs and CDGs. For comparison, chromatin enrichment of misregulated genes in Lamin-B knockdown in Kc cells and adult FB is shown. Percentage of genes in each category is labeled in each box, and the total number of genes analyzes is listed on top. The color of each box corresponds to the level of significance of the enrichment FETs, wherein blue indicates depletion and red indicates enrichment (*<0.05, **<0.01, ***<0.001, ****<0.0001). (C) Venn diagrams of overlap of EDGs with genes identified as testis clustered (Shevelyov *et al.* 2009). (D) Venn diagrams showing overlap of up- and downregulated CDGs with misregulated genes resulting from Lamin-B knockdown (KD) in Kc cells and in the adult fat body.

We reasoned that Lamin-B might also contribute to the repression of EDGs. This prediction is based upon previous studies demonstrating that the Drosophila B-type lamin contributes to gene silencing in multiple cell types (Shevelyov *et al.* 2009; Kohwi *et al.* 2013; Chen *et al.* 2014; Nazer *et al.* 2018; Shevelyov and Ulianov 2019). Two complementary analyses were used to address the role of Lamin-B in EDG repression. First, we overlapped the set of upregulated EDGs with genes upregulated upon Lamin-B knockdown in either Kc cells (Nazer *et al.* 2018) or in adult fat bodies (Chen *et al.* 2014). Whereas no significant overlap is found between upregulated genes in the two lamin knockdown backgrounds (*P = *0.91; Figure  4A), significant overlap was found between the upregulated EDGs and the Kc upregulated genes (17%; 27/157, *P =* 0.003) but not the fat body upregulated genes (15%; 23/157, *P = *0.08). These observations indicate that Lamin-B might contribute to EDG repression. Even so, the majority of EDGs remain unaffected by Lamin-B loss. Because transcriptional derepression requires both loss of a repressor and presence of an activator, we suggest that the limited number of shared genes in the Lamin-B and EDG datasets might reflect differences in activators present in the three cell types. Alternatively, emerin might confer lamin-independent repression. Second, we characterized the type of chromatin associated with EDGs. Five principal chromatin types have been defined based on protein composition (Filion *et al.* 2010). Genome-wide assessment of EDG-associated chromatin established that EDGs are significantly enriched in red chromatin (Figure  4B), a developmentally poised chromatin type that includes genes repressed by Lamin-B (Figure 4B;Nazer *et al.* 2018). Taken together, properties of EDGs suggest a possible partnership of emerin and Lamin-B in the silencing of transcriptionally poised genes in the ovary.

Transcriptional repression of clustered testis-specific genes in nontestis tissues requires Lamin-B (Shevelyov *et al.* 2009; Nazer *et al.* 2018). We wondered whether EDGs included clustered testis genes. First, we examined the genome-wide distribution of EDGs, finding no evidence of clustering (Supplementary Figure S3). These observations imply that EDGs do not reside in coregulated expression domains or localize within domains near centric heterochromatin. Second, we considered EDG overlap with an estimated 608 testis genes that reside in large and small clusters (Shevelyov *et al.* 2009). Comparison of these datasets revealed no significant overlap between EDGs and testes gene clusters [1% (2/157), *P = *0.99; Figure  4C], indicating that emerin is not required for repression of clustered testis genes in the ovary.

### CDGs are misregulated upon *lamin* knockdown in somatic tissues

CDGs were defined as *emerin* misregulated genes whose expression was restored in *chk2, emerin* double mutants (Figure  3C). To explore the properties of CDGs, we focused on upregulated CDGs (3526 genes) because this gene set is not confounded by changes in gene expression resulting from blocked differentiation, as occurs for downregulated genes. Notably, most (88%,1962/2219) genes upregulated upon *baf* GLKD correspond to CDGs (Figure  3C), consistent with Chk2 activation in *baf* mutants (Duan *et al.* 2020b).

We defined the chromatin context of CDGs. We found that CDGs are enriched in developmentally regulated chromatin, including red and blue chromatin (defined by association with the Polycomb group repressive complex, Figure  4B). As loss of Lamin-B altered expression of genes in similar chromatin types (Figure  4B), we assessed whether loss of Lamin-B caused upregulation of CDGs. To this end, we overlapped the set of upregulated CDGs with genes upregulated upon Lamin-B knockdown in either Kc cells (Nazer *et al.* 2018) or in adult fat bodies (Supplementary Table S2; Chen *et al.* 2014). Whereas the two lamin knockdown backgrounds do not show a significant overlap of upregulated genes (10%, *P = *0.91; Figure  4D), significant overlap was found with upregulated CDGs and both Kc upregulated genes (35%; 496/1406, *P *<* *1.4e−11) and fat body upregulated genes (36%; 498/1376, *P *<* *1.0e−13). The striking overlap between CDGs and Lamin-B upregulated genes suggests that NL dysfunction might commonly activate Chk2.

### CDGs identify potential targets of Chk2

**Figure 5:**
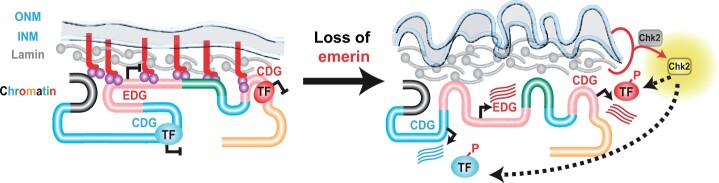
Dysfunction of the NL alters gene expression. Extensive LADs reside near the NL. These domains are enriched in repressive chromatin marks, including green (HP1a marked), blue (PcG marked), and black (Histone H1 marked), as well as the developmentally poised red chromatin (Histone deacetylases). This contrasts with active yellow chromatin (H3K36me3) that localizes away from the NL. Chromatin domains are tethered to the NL through multiple protein associations, including LEM-D proteins (red) embedded in the NL and BAF (purple dimer). In the absence of LEM-D proteins, NL structure and genome organization changes, effects that alter gene expression. Our data identify two classes of genes whose expression is affected upon emerin loss, EDGs, and CDGs. Upregulation of EDGs is Chk2 independent. EDGs are enriched in red chromatin, suggesting that upregulation of these genes results from loss of tethering resident domains to the NL. Upregulation of CDGs is Chk2 dependent. These genes are enriched in blue and red chromatin, suggesting that downstream targets of activated Chk2 might include phosphorylation sensitive TFs, wherein phosphorylation changes their function, resulting in the misexpression of genes in these domains.

We reasoned that CDGs might reveal TFs whose function is influenced by activated Chk2. For this reason, we submitted the set of upregulated CDGs to FlyEnrichr to identify whether any TF binding sites were over-represented in the dataset. Several significantly over-represented TFs were identified (Table  2). These TFs include Trithorax like (Trl also known as GAGA factor), a multi-functional sequence-specific DNA-binding protein that has roles in nucleosome organization, higher-order chromatin structure, as well as activation and repression of transcription (Chetverina *et al.* 2021). Trl-binding sites are commonly found in Polycomb response elements (Chetverina *et al.* 2021) and enriched in red chromatin (Filion *et al.* 2010), consistent with our findings that CDGs are distributed in red and blue chromatin. Notably, phosphorylation reduces Trl DNA binding (Bonet *et al.* 2005), suggesting that Trl function might be affected by activated Chk2 kinase. A second TF with significant enrichment was Suppressor of Hairy-wing [Su(Hw)]. This zinc-finger (Zn-F) transcriptional repressor is required for oogenesis (Klug *et al.* 1968, 1970; Baxley *et al.* 2011), with its central fertility function linked to transcriptional repression of the germ cell-specific *RNA-binding protein 9* (*Rbp9*) gene (Soshnev *et al.* 2013). Strikingly, this key Su(Hw) target gene is an upregulated CDG. DNA binding by Su(Hw) depends on three essential DNA-binding ZnFs that are separated by the conserved linker peptide TGEKP (Baxley *et al.* 2017). As global phosphorylation of this linker in other ZnF proteins disrupts DNA binding (Rizkallah *et al.* 2011), we suggest that activated Chk2 might phosphorylate the TGEKP in Su(Hw), thereby inhibiting its DNA binding and causing derepression of target genes. Indeed, we find that 40% (31/78, *P *=* *2.4e−03; Soshnev *et al.* 2013) of Su(Hw) repressed ovary target genes are CDGs. Of the five enriched identified TFs (Table  2), only Su(Hw) carries the conserved TGEKP in its DNA binding domain. Taken together, we propose that Chk2 activation might disrupt TF function through phosphorylation, resulting in altered gene expression (Figure  5). Further experiments are needed to determine whether the implicated TFs are direct targets of Chk2.

**Table 2:** Transcription factor enrichment of Chk2-dependent genes

Transcription factor	Adjusted *P*-value
Trithorax like (GAGA factor, GAF)	1.6e−111
Caudal	6.2e−79
Suppressor of Hairy-wing	1.2e−47
Mef2	4.2e−47
NELF-B	1.9e−38

### Concluding remarks

RNA profiling of RNA isolated from *emerin* and *baf* mutant ovaries uncovered a shared set of NL-regulated genes. Integrating these data with the RNA-seq profile of *chk2, emerin* double mutants established that Chk2 drives global misregulation of gene expression caused by NL dysfunction. Furthermore, this analysis identified two classes of misregulated genes, a small class of EDGs and a large class of CDGs. EDGs are broadly distributed in the genome (Supplementary Figure S3). Only upregulated EDGs show significant overlap with genes misexpressed upon BAF depletion (Figure  3C), suggesting that EDG repression involves the partnership between emerin and BAF. CDGs are also widely distributed in the genome and show near complete overlap with genes upregulated upon *baf* GLKD (Figure  3C), consistent with Chk2 activation in *baf* mutants (Duan *et al.* 2020b). Notably, Lamin-B loss in two additional cell types shows significant upregulation of CDGs (Figure  4D). Taken together, our data support a model wherein dysfunction of gene expression in *NL* mutants results from alteration in two regulatory inputs, one from the NL and a second from activated Chk2 (Figure  5). Furthermore, our studies suggest that Chk2 might contribute to altered gene expression found in other *NL* mutant backgrounds.

## Data availability

Strains are available upon request. RNA-seq data are submitted to Gene Expression Omnibus under the accession number GSE168062. Supplemental Material is available at figshare: https://doi.org/10.25387/g3.17049932.
